# Transcatheter Tricuspid Valve Interventions After Heart Transplantation: Walking the Tight Rope

**DOI:** 10.1016/j.jaccas.2026.107047

**Published:** 2026-03-04

**Authors:** Prashant Dwivedi, Kush Mehrotra

**Affiliations:** Department of Interventional Cardiology, Eternal Hospital, Jaipur, Rajasthan, India

**Keywords:** cardiac transplant, tricuspid valve, valve replacement

**Figure:**
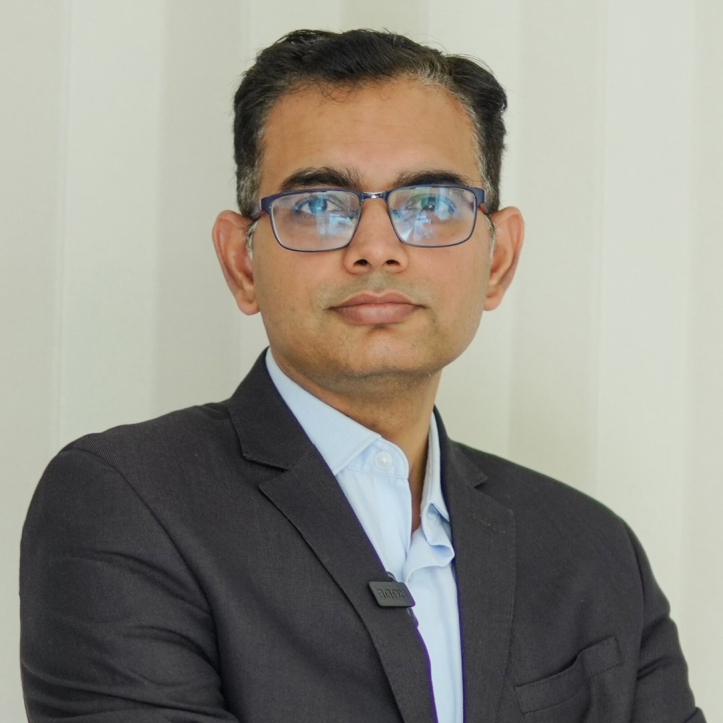

Heart transplantation (HTx) is a destination procedure for selected patients with end-stage heart failure. The enhanced median survival of HTx patients is up to 13 years,^1^ and incorporation into the HTx program of marginal donors—those with elderly status or associated comorbidities including diabetes, hypertension, left ventricular hypertrophy, mild coronary artery, or valvular heart disease—has led to the emergence of late complications after HTx such as valvular and conduction system disease.^2^

For HTx patients, with their unique physiology and relatively limited life span, quality of life takes precedence over quantity. With the increase in number and age of HTx patients, surgical or interventional corrections of allograft is expected to rise.

The prevalence of significant tricuspid regurgitation (TR) ranges from 19% to 84%, making it the most common valvular abnormality, with up to 14% of HTx recipients developing severe TR at 10 years.^3^^,^^4^ Functional TR typically develops early after HTx and depends on several factors such as surgical technique (biatrial anastomoses), frequent allograft rejection, pre-existing pulmonary hypertension, or donor size mismatch. On the other hand, anatomical TR presents late and is more often attributed to endomyocardial biopsy (EMB).^5^

History of sternotomy, associated comorbidities, and ongoing immunosuppressive therapy makes any future intervention in this group challenging, often with prohibitory operative risks. Medical management forms the mainstay, and invasive treatment is usually reserved for refractory patients despite optimal medical therapy.

TR in HTx recipients differs significantly from that in the general population owing to the alterations in atrial geometry and ventricular function related to rejection episodes or graft adaptation, however its echocardiographic assessment is based on guidelines established for the general population. In this context, cardiac magnetic resonance (CMR) overcomes these limitations, avoids geometrical assumptions, and provides tissue characterization. These insights are vital for tailoring therapeutic interventions and optimizing outcomes in patients with TR.^5^

For the prevention of functional TR, prophylactic tricuspid annuloplasty (TA) has been attempted, which effectively prevents severe TR but does not offer significant long-term hemodynamic benefits. Moreover, TA in HTx is linked to a higher occurrence of complete heart block, resulting in more frequent pacemaker implantations.^6^ Prophylactic TA during HTx by eliminating natural pressure relief function of TR can increase the risk of right heart failure, particularly when pulmonary arterial hypertension is present. Surgical tricuspid valve (TV) replacement with a bioprosthesis is preferentially performed, as TV repair may be susceptible to failure after repeated EMBs, and mechanical prosthesis precludes future EMBs.^7^

Currently, there are 4 modalities of transcatheter treatment available for TR: tricuspid annuloplasty (TA), heterotopic bicaval valve implantation, transcatheter edge-to-edge repair (TEER), and transcatheter tricuspid valve replacement (TTVR). There are many case reports or small case series of TEER after HTx but very few case reports of TTVR in post-HTx patients. In this issue, Orzalkiewicz et al^8^ have demonstrated successful TTVR after HTx with sustained short-term clinical benefits.

Complex TV anatomy in HTx recipients poses significant technical hinderances to successfully practice transcatheter interventions routinely on a large scale. TEER (Figure 1) shows good short-term outcomes, but TR is rarely entirely abolished and recurrence has been reported. Damaged or torn leaflets in anatomical TR may also make TEER impractical. Additionally, future EMBs become difficult after TEER, and a biopsy may potentially predispose to failure of the repair. Heterotopic bicaval valve implantation may be feasible after HTx independent of surgical technique (biatrial vs bicaval anastomosis). Nevertheless, this is a palliative treatment that does not address the TR or right ventricular function, and it should be sparingly considered.

**Figure 1:**
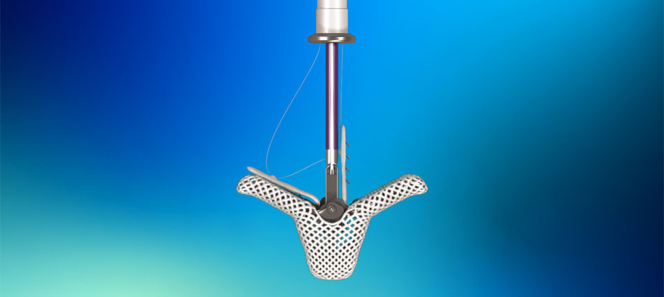
TriClip for Transcatheter Edge-to-Edge Repair

TTVR is a promising therapeutic strategy for severe TR irrespective of underlying etiology. Evoque (Figure 2) (Edwards Lifesciences) is the only platform approved for severe TR in the general population, and it offers near-complete resolution of TR. HTx recipients, however, were excluded from the TRISCEND trial. TTVR offers hope, does not preclude future EMBs, and is less prone to damage during EMB. Nevertheless, TTVR's high efficacy accompanies a higher rate of permanent pacemaker implantation (PPM) (24%) and an increased bleeding risk (>10%), predominantly of gastrointestinal origin, which complicates antithrombotic management,^9^ when compared with TEER. HTx recipients, individualistically, have high thrombotic as well as bleeding risk.

**Figure 2:**
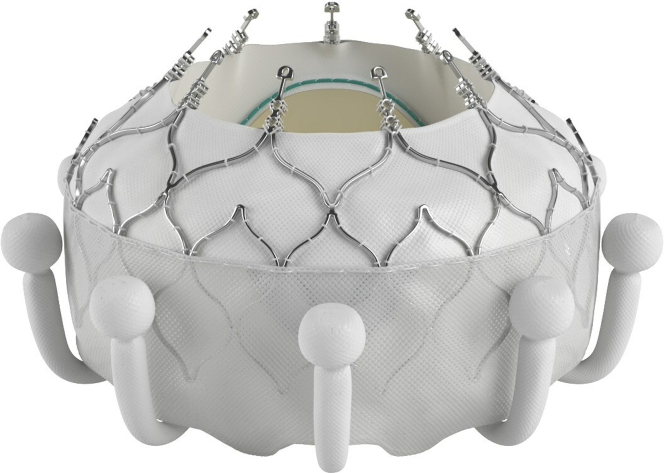
Evoque Bioprosthetic Valve for Transcatheter Tricuspid Valve Replacement

Thus, to choose between TEER or TTVR in post-HTx patients, certain issues demand consideration, such as the need for future EMBs, the balance between thrombotic versus bleeding risk, the need for anticoagulation, and the future need for PPM (Figure 3). Annulus- or anchor-sparing technology along with noninvasive detection of graft rejection maybe the way forward. The era of transcatheter valve therapy in post-transplant patients has just begun, but substantial challenges persist before its wider safe implementation.

**Figure 3:**
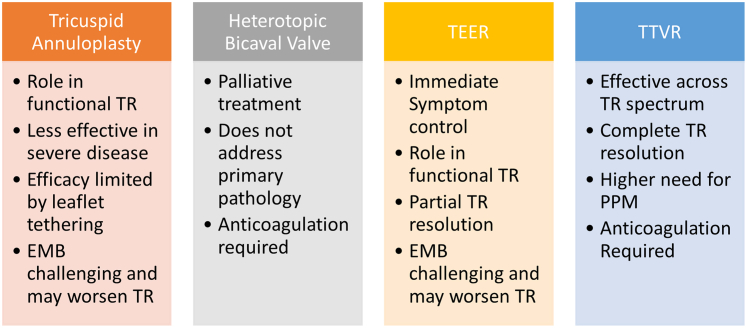
Comparative Table Summarizing the Pros and Cons of Various Transcatheter Treatment Options for Severe TR After Heart Transplantation EMB = endomyocardial biopsy; PPM = permanent pacemaker; TEER = transcatheter edge-to-edge repair; TR = tricuspid regurgitation; TTVR = transcatheter tricuspid valve replacement.

## Funding Support and Author Disclosures

The authors have reported that they have no relationships relevant to the contents of this paper to disclose.

## References

[1] Khush K.K., Cherikh W.S., Chambers D.C. (2019). The International Thoracic Organ Transplant Registry of the International Society for Heart and Lung Transplantation: Thirty-sixth adult heart transplantation report—2019; focus theme: donor and recipient size match. J Heart Lung Transpl.

[2] Ahmad K., Terkelsen C.J., Terp K.A. (2016). Transcatheter aortic valve implantation in a young heart transplant recipient crossing the traditional boundaries. J Thorac Dis.

[3] López-Vilella R., Paniagua-Martín M.J., González-Vílchez F. (2022). Prevalence of tricuspid regurgitation after orthotopic heart transplantation and its evolution in the follow-up period: a long-term study. Transpl Proc.

[4] Wong R.C.C., Abrahams Z., Hanna M. (2008). Tricuspid regurgitation after cardiac transplantation: an old problem revisited. J Heart Lung Transplant.

[5] Alyaydin E., Gotschy A., Parianos D. (2025). Tricuspid regurgitation after heart transplantation: where innovation and intervention are in hibernation. Heart Fail Rev.

[6] Rubin G.A., Sanchez J., Bayne J. (2019). Conduction abnormalities associated with tricuspid annuloplasty in cardiac transplantation. ASAIO J.

[7] Cuko B., Baudo M., Busuttil O. (2024). Outcomes of tricuspid valve prostheses after heart transplantation: a systematic review. Heart Fail Rev.

[8] Orzalkiewicz M., Palmerini T., Biagini E. (2026). Successful transcatheter tricuspid valve replacement 30 years after heart transplantation. JACC Case Rep.

[9] Hahn R.T., Makkar R., Vinod V.H. (2025). Transcatheter valve replacement in severe tricuspid regurgitation. New Engl J Med.

